# Fifteen years of ChEMBL and its role in cheminformatics and drug discovery

**DOI:** 10.1186/s13321-025-00963-z

**Published:** 2025-03-10

**Authors:** Barbara Zdrazil

**Affiliations:** European Molecular Biology Laboratory, European Bioinformatics Institute (EMBL-EBI), Wellcome Genome Campus, Hinxton, Cambridgeshire, CB101SD UK

**Keywords:** Open data, ChEMBL, FAIR, Cheminformatics, Drug discovery

## Abstract

In October 2024 we celebrated the 15th anniversary of the first launch of ChEMBL, Europe’s most impactful, open-access drug discovery database, hosted by EMBL’s European Bioinformatics Institute (EMBL-EBI). This is a good moment to reflect on ChEMBL’s history, the role that ChEMBL plays in Cheminformatics and Drug Discovery as well as innovations accelerated using data extracted from it. The review closes by discussing current challenges and possible directions that need to be taken to guarantee that ChEMBL continues to be the pioneering resource for highly curated, open bioactivity data on the European continent and beyond.

**Supplementary Information:**

The online version contains supplementary material available at 10.1186/s13321-025-00963-z.

## A short history on the organization of bioactivity data

At the beginning of the twenty-first century, the drug discovery community was facing a situation of rapid growth of large-scale bioactivity data in the open domain. Several interrelated factors were driving this fast-paced development, including the rise of high-throughput screening (HTS) techniques and other technological advances such as chemical syntheses automation, the increasing emphasis on pre-competitive collaboration and data sharing, and the growing recognition of the value of open data for accelerating drug discovery. Moreover, the Sanger Centre (now the Wellcome Sanger Institute, located at the Wellcome Genome Campus) in Hinxton, England, was one of the most significant contributors to the Human Genome Project from 1990 to 2003 (they sequenced about a third of the human genome) [1] which led to increased knowledge about the genetics of (potential) drug targets but also a growing demand to study targets experimentally.

These developments together spurred the need to collect, curate, standardise, and store bioactivity data in an organised way in the early 2000’s. At that point in time, Inpharmatica Ltd., a UK-based biotech firm which was acquired by Galapagos NV in 2006 focused their data collection and storage on small molecules, biological targets, and their interactions, aiming to create a resource that could inform better drug design and target selection. Their product “StARlite” originated from the vision of John Overington and his team [2] was subsequently transferred to EMBL-EBI, where it found a new home and received funding from the Wellcome trust to launch ChEMBL as an open-access database; for the first time in October 2009 [3, 4].

As inherent in its original name (“StARlite”), Structure–Activity Relationship (SAR) data extracted from Medicinal Chemistry Literature was the focus at that time. The data was extracted from 12 different journals (*Eur. J. Med. Chem., Nat. Biotechnol., Proc. Natl. Acad. Sci. USA, Bioorg. Med. Chem., J. Biol. Chem., Antimicrob. Agents Chemother., Drug Metab. Dispos., Science, Bioorg. Med. Chem. Lett., J. Med. Chem., J. Nat. Prod., Nature*) with bioactivity data originating from ~ 26 thousand documents, covering ~ 330 thousand different assays, ~ 5400 targets, and ~ 440 thousand chemical compounds. The first version of ChEMBL was composed of only 15 public-facing tables [5].

It was, however, anticipated early on that ChEMBL would quickly grow into a resource with more diverse data types capturing bioactivity data from not only scientific literature but also from direct data depositions and from the addition of data partitions from other public databases. ChEMBL 03 (released in April 2010) introduced the source column (SRC_ID) in the assays table “to capture the fact that, in future, ChEMBL may capture data from sources other than Scientific Literature” as stated in the release notes [6].

In fact, from ChEMBL 04 (released in May 2010) onwards, the plan to incoorporate more diverse data sources was implemented, with the first direct data depositions being in the area of neglegted tropical diseases (NTDs) such as Plasmodium falciparum screening data from GSK, Novartis/GNF and St. Jude Children's Research Hospital [7]. In parallel, the ChEMBL-NTD server was launched to provide early open access to NTD screening data, usually in a raw, uncurated data format. Deposition to this platform turns the data set into a citable item even before publication of the curated data set in ChEMBL [8].

**Fig. 1:**
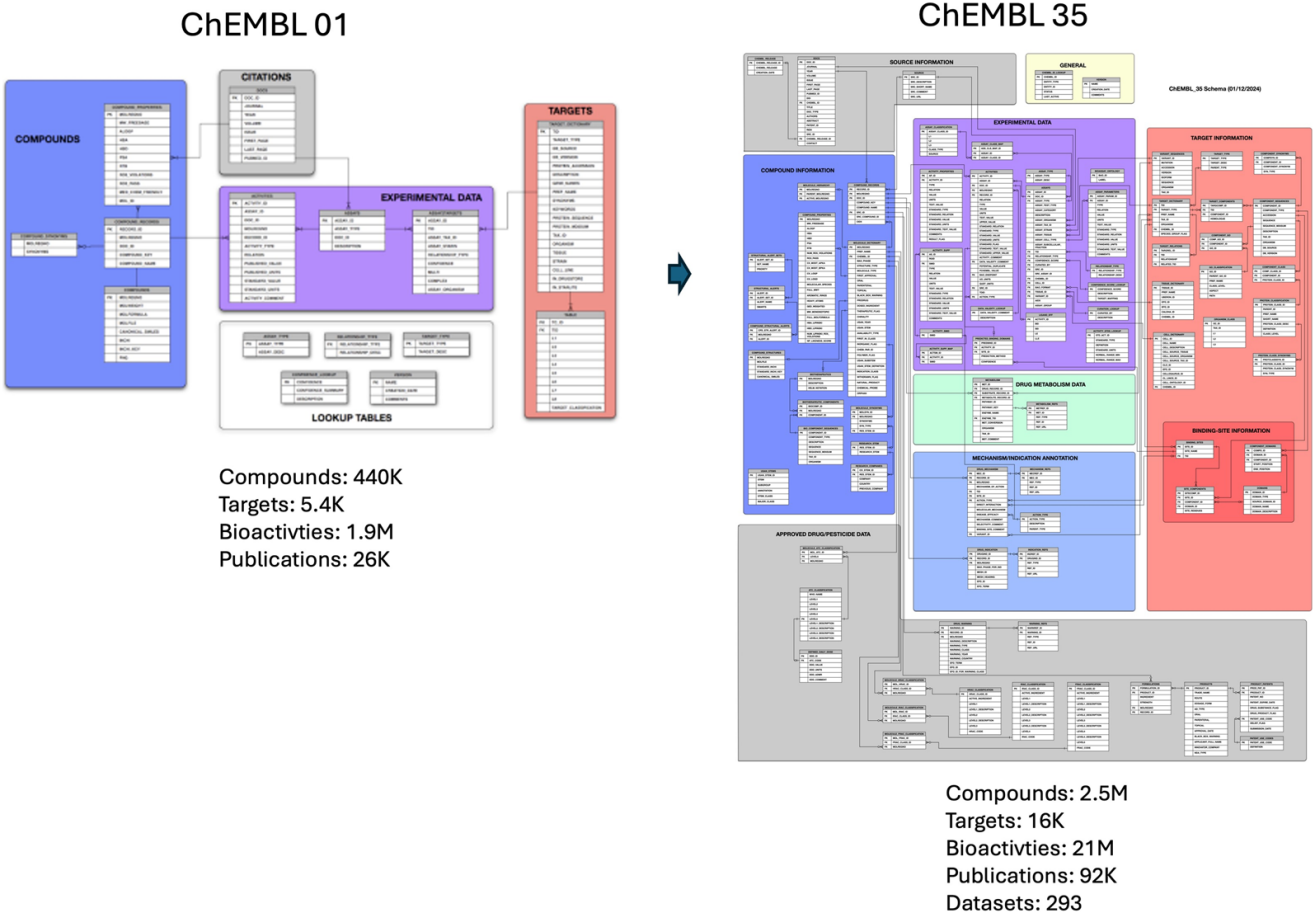
Comparison of database schemas for ChEMBL 01 vs. ChEMBL 35. Higher resolution images for both entity-relationship diagrams (ERDs) can be found as part of the respective ChEMBL release notes [29] as well as in Supplements (Supplementary Figs. 1 and 2)

The ChEMBL database schema has significantly changed over time (Fig. 1) to be able to accomodate additional data types, but also to promote a FAIR (Findable, Accessible, Interoperable, Reusable) representation of the data entities. The first major schema changes have been performed for ChEMBL 08 (November 2010) and 09 (February 2011). ChEMBL entities received unique identifiers for compounds, targets, assays and documents in the form 'CHEMBL123456'. From ChEMBL 08 onwards, the MOLECULE_HIERARCHY table allowed to properly store parent-salt relationships for chemical compounds and the MOLECULE_DICTIONARY included many new fields which serve to describe certain drug properties (e.g., MOLECULE_TYPE, FIRST_APPROVAL, BLACK_BOX_WARNING, PRODRUG, DOSED_INGREDIENT, THERAPEUTIC FLAG) for the newly added data on biotherapeutic drugs.

The first data deposition of a partition of another public database into ChEMBL happened in the course of the ChEMBL 10 release (June 2011): a subset of data from the PubChem BioAssay database, namely dose–response endpoints (e.g., IC_50_, K_i_, Potency) from confirmatory assays in PubChem [9], has been included. This was followed by data from the Guide to Receptors and Channels [10] and some first toxicity datasets, like the Open TG-GATEs dataset [11], and some public data sets for phospholipidosis and hepatotoxicity (extracted from scientific literature) in ChEMBL 11 (August 2011). The growing amount of data made it necessary to structure the organism information of the targets by introducing the ORGANISM_CLASS table, based on the NCBI taxonomy [12]. In addition, an effort started to automate data standardisation protocols to a greater extend from ChEMBL 12 (December 2011) onwards, e.g., for measurement types, values and units. Due to a growing number of targets, target identifiers for non-single protein targets (such as protein complexes or protein families) have been introduced at the same time.

A big push for increasing the number of toxicity data sets, annotated drug data sets, as well as data for NTDs happened with ChEMBL 14 and 15 in 2012/13 (with DrugMatrix in vitro pharmacology assays [13], in vivo data from Open TG-Gates [14], USAN applications [15] and INNs [16], the MMV Malaria box [17], GSK Tuberculosis Screening Data, and Harvard Malaria Screening Data etc. being deposited).

Another landmark was certainly the introduction of the pChEMBL value with ChEMBL 16 (May 2013) which allows a number of roughly comparable measures of half-maximal response concentration/potency/affinity to be compared on a negative logarithmic scale [18]. At the same time, the database was also made available in RDF format for the first time.

ChEMBL 17 (September 2013) for the first time made available information regarding the mechanism of action for FDA-approved drugs (stored in the DRUG_MECHANISM table). ChEMBL 18 (April 2014) made an effort to improve ontological mappings to, e.g., Cell Line Ontology [19], Experimental Factor Ontology (EFO) [20] and Cellosaurus Ontology [21]. Moreover, the BioAssay Ontology (BAO) [22] was used to map the BAO_ENDPOINT (e.g., IC_50_, K_i_) and assign the BAO_FORMAT (e.g., cell-based format, tissue-based format).

In ChEMBL 19 (July 2014) the content of ChEMBL was expanded to include more than 40 K compound records and 245 K bioactivity data points relevant to crop protection research (covering insecticides, fungicides and herbicides extracted from a number of different journals). Consequently, ChEMBL 20 (February 2015) introduced classification schemes for pesticides (fungicides, herbicides, and insecticides) by Mechanism of Action (MoA) and chemical class.

Drug Indications for FDA approved drugs have been identified from a number of sources for ChEMBL 21 (March 2016), including Prescribing Information, ClinicalTrials.gov and the WHO ATC classification [23]. Mapping to both Medical Subject Headings (MeSH) disease identifiers and EFO disease identifiers guarantees maximum data FAIRness. Also, drug metabolism and pharmacokinetic (PK) data from a number of data sources was included for the first time. These included curated drug metabolism pathway data from a variety of literature sources, data extracted from FDA drug approval packages, as well as data extracted from the Journal Drug Metabolism and Disposition.

The scope of ChEMBL was further expanded in a collaborative effort with the NIH-funded Illuminating the Druggable Genome (IDG) project [24] by including bioactivity data for understudied targets from selected SureChEMBL patents; for the first time in ChEMBL 23 (May 2017) [25]. To date, ~ 57 K compounds measured on 1673 distinct targets (~ 184 K bioactivities) are reporting bioactivity data extracted from SureChEMBL patents.

ChEMBL 24 (June 2018) included a major reformatting of supplementary data tables (ACTIVITY_PROPERTIES table, ACTIVITY_SUPP table) which made it possible to store complex assays against one individual assay identifier, e.g., when measurements at different time points or at different compound concentrations have to be recorded (e.g., DrugMatrix and Open TG-GATEs bioactivity data). ChEMBL 25 (March 2019) introduced the in vivo assay classification schema (ASSAY_CLASSIFICATION table) consisting of a three-level classification [26].

A new in silico target prediction tool based on conformal prediction was provided with ChEMBL 26 (March 2020), replacing an older tool [27]. ChEMBL 27 (May 2020) was a special COVID-19 release, incorporating data from eight drug repurposing papers, which tested the efficacy of approved drugs, clinical candidates and other selected compounds against SARS-CoV-2 infection/replication in cell-based assays.

ChEMBL 28 (February 2021) was the first release that included chemical probe data and a chemogenomic library deposited as part of the EUbOPEN project [28].

The latest releases of ChEMBL, versions 32–35, included a few schema changes, to introduce new features and deprecate some legacy features. These changes included an update of the algorithm to calculate natural product-likeness, the addition of flags for natural products, chemical probes, and orphan drugs. The new ACTION_TYPE field as part of the ACTIVITIES table (released in CHEMBL 33) provides additional detail on the mode of action of tested compounds in the specific assay setup. The information is still sparse but will be populated for more bioactivity endpoints in future releases. Furthermore, an effort was undertaken to improve data provenance by time stamping documents of deposited data sets. By introducing the new CHEMBL_RELEASE table the CREATION_DATE can now more easily be retrieved for each document. The very recent release of ChEMBL 35 (December 2024), introduced additional new features to increase data provenance and also FAIRness: the source of every document is now described in more detail in the SOURCE_COMMENT and the new CONTACT field in the DOCS table offers the option to associate data sets with a primary point of contact (ideally ORCID IDs or other stable identifiers of researchers).

More detailed information on updates to the ChEMBL database in the past ~ 4 years are described in the latest database update paper in Nucleic Acids Research [30].

Most of the modifications applied to the database schema described in this section are an effect of an expanded scope of the ChEMBL database or else they resulted from the availability of improved features and software to replace outdated tools. As illustrated in Fig. 1, the database schema has expanded in size from 15 (ChEMBL 01) to currently 78 tables (ChEMBL 35). While the ChEMBL database is nowadays able to accommodate bioactivity data from a wide variety of different data types and disease modalities, the same improvements have also led to a greater workload for the ChEMBL team when it comes to loading and curating data sets with novel data types and/or complex metadata information.

## The role of ChEMBL in advancing cheminformatics and drug discovery

ChEMBL's contributions to the field of cheminformatics and drug discovery extend beyond providing a valuable data resource for research; it was also fundamental in catalysing method development in cheminformatics and related fields as well as in spurring more holistic approaches by offering a multi-target view on drug discovery. Furthermore, over the years ChEMBL spurred the discussion and set standards for FAIR and open data distribution [31, 32] and therefore serves as a role model for other open data providers. The latter influences related research fields such as subdomains of bioinformatics that focus on biomedical ontology development.

While the intend of this commentary is not to provide a comprehensive literature review of publications that report the use of ChEMBL in their research, it tries to summarise a few impactful studies that were enabled/facilitated by the existence of ChEMBL.

**Fig. 2:**
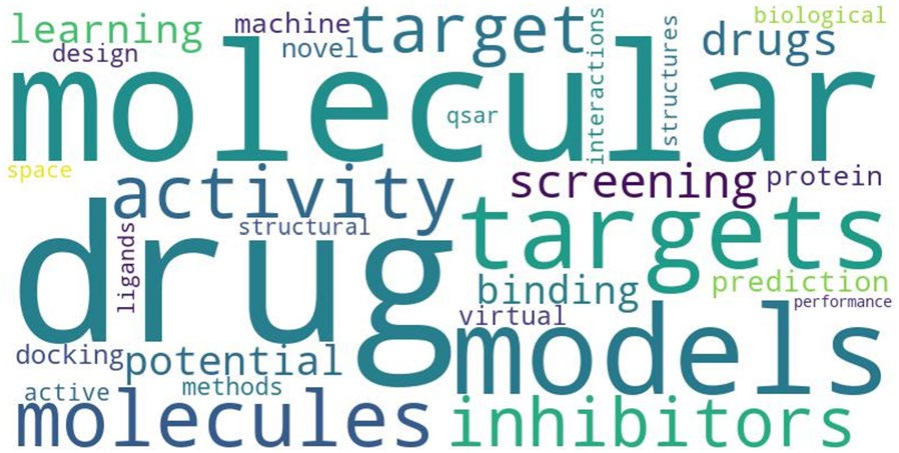
Wordcloud produced by using the PubMed API to retrieve articles between 2010 and 2024 (912 papers in total) that mention “ChEMBL” in either the title or abstract (using the nlkt and wordcloud packages in python; number of common keywords extracted = 30). These are the most common keywords identified: ('drug', 1105), ('molecular', 824), ('models', 812), ('targets', 635), ('molecules', 606), ('activity', 591), ('inhibitors', 590), ('target', 565), ('screening', 536), ('drugs', 522), ('potential', 495), ('learning', 476), ('binding', 440), ('prediction', 433), ('protein', 390), ('machine', 377), ('docking', 360), ('novel', 348), ('virtual', 330), ('design', 297), ('methods', 291), ('active', 259), ('space', 246), ('structural', 243), ('biological', 242), ('qsar', 238), ('structures', 237), ('ligands', 234), ('interactions', 230), ('performance', 223)

**Fig. 3:**
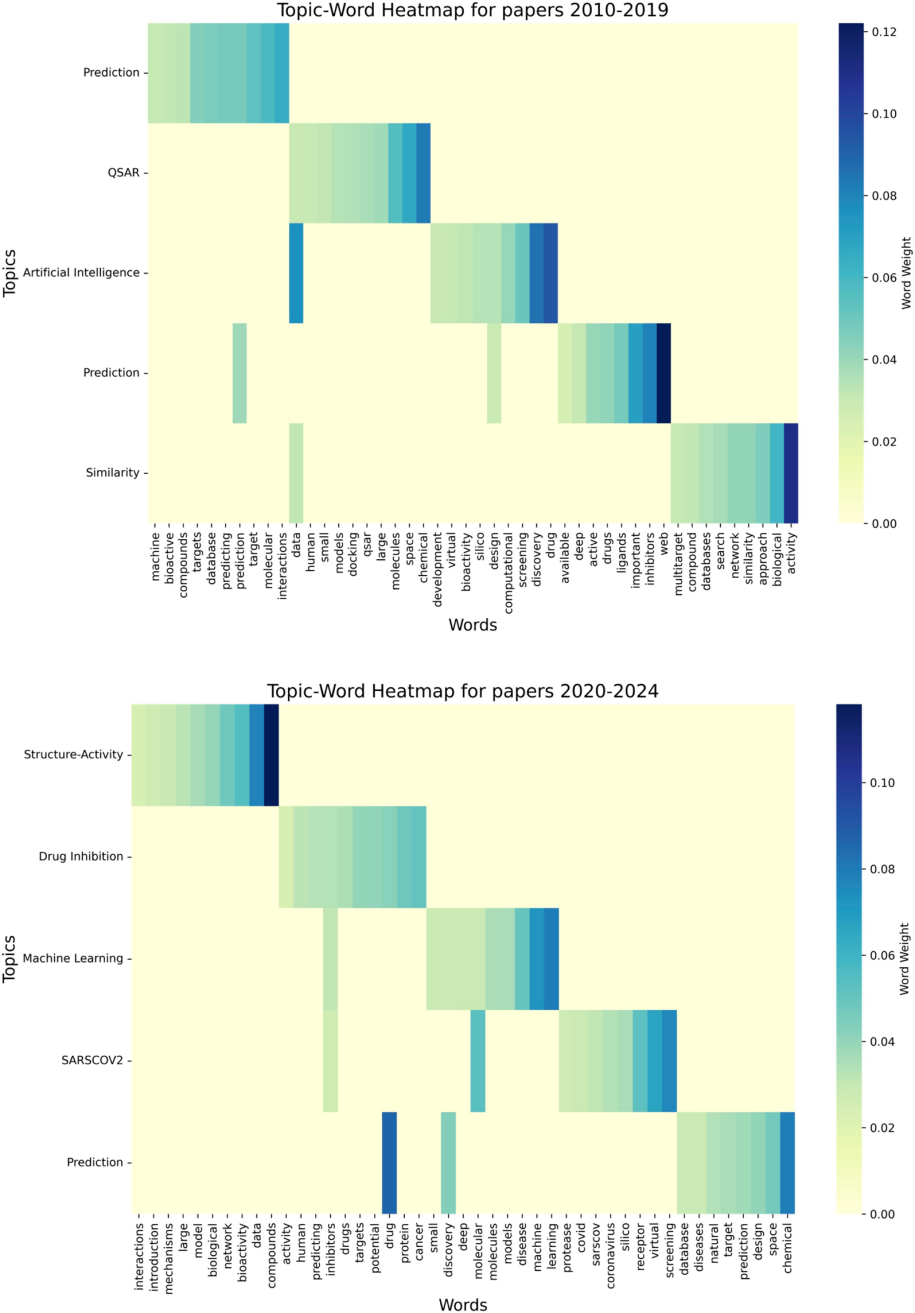
Heatmaps showing the relationships between the identified topics and the top words associated with those topics, which were derived from topic modeling based on articles in PubMed that contain the term “ChEMBL” in either title or abstract. Darker colour indicates a higher word weight (higher prevalence). Words within a topic are ordered by increasing word weight from left to right. The left heatmap is based on 421 articles published between 2010 and 2019; the right heatmap is based on 511 articles published between 2020 and 2024. Topic modelling was performed using Latent Dirichlet Allocation (sklearn package in python) by retrieving 5 topics and 10 words, respectively, for each time period. Each topic is represented by a list of words initially, with weights indicating how important each word is to the topic. Further a Sentence Transformer model (sentence-transformers library in python) was used to match a predefined set of single representative terms using word embeddings and cosine similarity

Before delving into particular publications, some high-level biblometric analyses were performed in order to capture the frequency/importance of specific topics that papers published by using data from ChEMBL investigate. To this end, the PubMed API was leveraged to retrieve all articles that mention the term “ChEMBL” in either its title or abstract (or both). Frequent terms of these articles are visualised in the form of a wordcloud in Fig. 2, giving some hints on potential areas of use of ChEMBL data, such as for studying molecular interactions, investigating drugs, targets, or inhibitors. In terms of methods, we see the prevalent use of terms such as docking/virtual screening, machine learning, QSAR (quantitative structure–activity relationship). A more sophisticated analysis uses a topic modelling approach to cluster words on the basis of their prevalence and cosine similarity into a defined number of topics. As seen in Fig. 3, topics slightly vary for the different time periods, which were selected in order to have a comparable number of papers for each period, respectively (2010–2019 and 2020–2024). While the topic labels have been assigned on the basis of semantic similarity, they were pre-defined by using a list of frequent topics in drug discovery and cheminformatics in the past 15 years. Thus, the exact meaning of those labels should not be over-interpreted but rather the list of frequent words for each topic shall serve as basis for interpretation of interest in a specific research field. Not surprising is the increased interest in infectious diseases, specifically research on SARS-CoV-2 in more recent years.

One of the most significant impacts of ChEMBL in cheminformatics has been its role in supporting QSAR modelling. By offering structured and well-curated data on chemical compounds and their biological activities, ChEMBL serves many researchers as a source for generating predictive in silico models for specific protein targets, or for a plethora of multiple targets simultaneously (the latter is often referred to as multi-target QSAR). Such investigations also spurred methodological developments in the fields of cheminformatics and related fields. Examples include, e.g., a study exploring the use of taxonomy-based multi-task learning to improve multi-target QSAR models for drug discovery, particularly by leveraging knowledge transfer between similar targets by Rosenbaum et al. [33]; a study by Lounkine et al. introducing a method termed “Chemotography”, which visualizes structure–activity relationships (SAR) in the context of complex biological pathways, offering a new paradigm for understanding drug interactions within biological systems [34]; and the SAR Matrix methodology by Ye Hu et al. which helps systematic extraction and analysis of large-scale SARs and exploration of mulitarget actvity spaces in chemogenomics [35].

The existence of ChEMBL also encouraged the development of new methods for data mining, predictive modeling, and machine learning. For these methods, the integration of bioactivity data from multiple data sources became increasingly important as mentioned in several studies over the years [36–38]. Also, the concept of integrating and utilising negative data (inactive bioactivity data) in predictive in silico models in order to enhance their accuracy was realised by leveraging ChEMBL data (among other sources) [39].

Notably, ~ 24% of all articles (222 papers) mentioning “ChEMBL” in their abstract or title, do also mention the term “machine learning”. There is a clear upward trend of such papers being published recently, with 29, 37 and 45 articles, respectively, published in the years 2022–2024. Apart from the use of ChEMBL data for generating machine learning (ML) models and testing new ML algorithms, large and highly curated data sets play an increasingly important role in benchmarking for the purpose of systematically evaluating and comparing the performance of algorithms. ChEMBL served in that way too as demonstrated by multiple papers [40–42]. ChEMBL’s bioactivity data was also used in studies combing ligand- and structure based molecular modelling, such as ML-based virtual screening approaches [43–46].

In computational toxicology, ChEMBL plays a significant role as well as data from ChEMBL offers manifold ways to explore toxicity. For instance, bioactivity data from ChEMBL for specific off-targets can serve to build predictive in silico models that can be used in the hit-to-lead or lead optimisation phases during drug development or as part of a regulatory submission process. Examples include models for human Ether-a-go-go Related Gene (hERG) [47] or hepatic Organic Anion Transporting Polypeptides (OATPs) [38]. By including mechanistic information from adverse outcome pathways (AOPs), one can also leverage information about molecular initiating events (MIEs) and extract bioactivity data for protein targets linked to MIEs as shown by Gadaleta et al. [48]. Predictive binary QSAR models built for those targets can be used as proxies for, e.g., organ-specific toxicities of chemicals.

Another way to make use of ChEMBL data for toxicity studies is to start from a chemical structure and query ChEMBL for potential (human) protein targets that might be affected by the chemical. In a study by Hong et al. [49] the ChEMBL-derived targets served as the foundation for further network toxicology and pathway enrichment analyses, which provided insights into biological processes and signaling pathways influenced by Bisphenol A.

It is worth noting, that the majority of assays in ChEMBL are of type “Functional” (830 K) and “Binding” (520 K), but ChEMBL 35 also contains 300 K and 60 K assays of type “ADME” and “Toxicity”, respectively. In vivo data in ChEMBL has been thoroughly curated and annotated with the animal disease model or phenotypic endpoint [50].

The availability of detailed chemical structure data in ChEMBL alongside with bioactivity measures and detailed information on protein targets also allows users to explore chemical and biological similarity of small molecules. These possibilities led to, e.g., advancements in the development of methods for chemical similarity measures [51–53], target prediction algorithms [54–56], the exploration of the concepts of polypharmacology [57, 58] and activity cliffs [59, 60], and the way how chemical space is navigated and visualised. Excellent reviews on these topics have been provided by, e.g., the research group of J.-L. Reymond [61, 62].

In the history of ChEMBL, the deposition of bioactivity data for neglected diseases has played an important role from the start. Academic research relies mostly on open data and can also afford to study commercially less attractive targets/diseases. Thus, several studies utilizing data from ChEMBL do also focus on neglected and tropical diseases, such us tuberculosis [63], dengue fever [64], or malaria [65].

## Conclusions

Over the past 15 years, ChEMBL has solidified its role as a pioneering database of highly curated and structured bioactivity data in the fields of cheminformatics and drug discovery. Its evolution, from the foundational StARlite database to its current form as ChEMBL 35, reflects its adaptability to scientific advancements and expanding data needs. ChEMBL’s impact extends beyond being a resource; it has catalyzed method development, inspired multidisciplinary research, and advanced the principles of FAIR data sharing.

ChEMBL’s influence is particularly notable in enabling predictive modeling, machine learning applications, computational toxicology, and computational drug discovery. Its data has empowered researchers to explore chemical space, design (safer) drugs, and address challenges in neglected disease areas. The continuous refinement of its schema and tools underscores its commitment to meeting the growing complexity of bioactivity data.

Looking forward, ChEMBL’s success depends on navigating challenges such as curating increasingly diverse datasets with an ever-expanding diversity of assays and experimental conditions as well as supporting new modalities in drug discovery. In future, even more accurate annotations of assays will be needed to make optimal use of ChEMBL for building large training sets for ML applications.

By maintaining its ethos of open-access collaboration and innovation coupled with very high standards for data curation, ChEMBL is poised to remain a leading resource for preclinical bioactivity data as well as clinical candidate and drug data globally, accelerating discoveries that bridge chemistry and biology for years to come.

## Supplementary Information

Supplementary Material 1: Fig. 1: Entity–relationship diagram for ChEMBL 01.

Supplementary Material 2: Fig. 2: Entity–relationship diagram for ChEMBL 35.

## Data Availability

No datasets were generated or analysed during the current study.

## Abbreviations

AOP: Adverse outcome pathway
BAO: BioAssay Ontology
EFO: Experimental factor ontology
EMBL-EBI: EMBL’s European Bioinformatics Institute
ERD: Entity-relationship diagram
FAIR: Findable, accessible, interoperable, and reusable
hERG: Ether-a-go-go related gene
HTS: High-throughput screening
IDG: Illuminating the druggable genome
ML: Machine learning
MeSH: Medical subject headings
MoA: Mechanism of action
MIE: Molecular initiating event
NTD: Neglegted tropical disease
PK: Pharmacokinetic
SAR: Structure–activity relationship
OATP: Organic anion transporting polypeptide
QSAR: Quantitative structure–activity relationship
