# Supplementary material for: Fifteen years of ChEMBL and its role in cheminformatics and drug discovery
Source: J Cheminform. 2025 Mar 10;17:32. doi: 10.1186/s13321-025-00963-z (PMC11895189; doi:10.1186/s13321-025-00963-z)
Supplement: Supplementary file 1 — Supplementary Material 1: Fig. 1: Entity–relationship diagram for ChEMBL 01. [file 13321_2025_963_MOESM1_ESM.pdf]

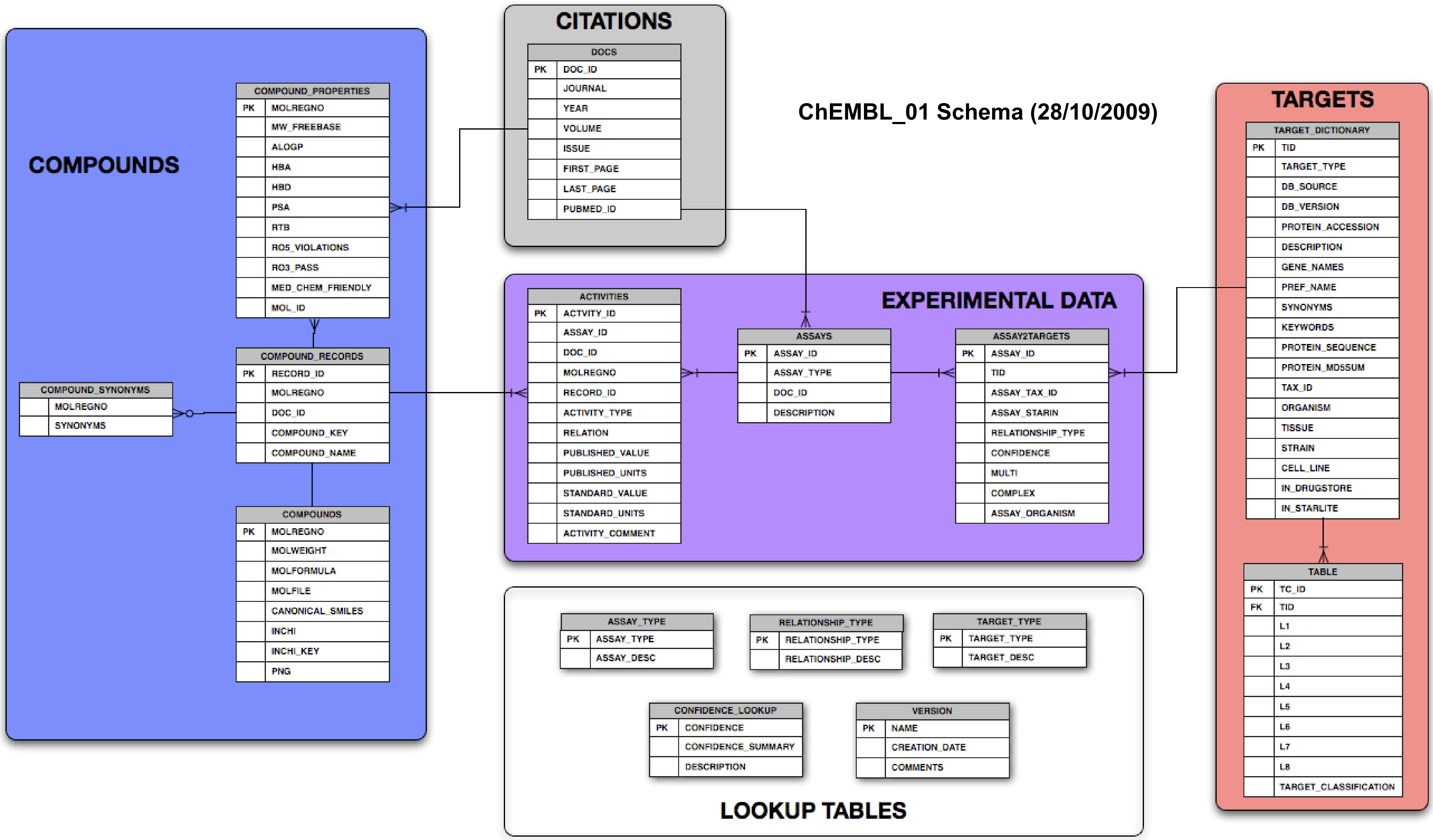
ChEMBL_01 Schema (28/10/2009)
COMPOUNDS
COMPOUND_PROPERTIES
PK MOLREGNO
MW_FREEBASE
ALOGP
HBA
HBD
PSA
RTB
RO5_VIOLATIONS
RO3_PASS
MED_CHEM_FRIENDLY
MOL_ID
COMPOUND_RECORDS
PK RECORD_ID
MOLREGNO
DOC_ID
COMPOUND_KEY
COMPOUND_NAME
COMPOUND_SYNONYMS
MOLREGNO
SYNONYMS
COMPOUNDS
PK MOLREGNO
MOLWEIGHT
MOLFORMULA
MOLFILE
CANONICAL_SMILES
INCHI
INCHI_KEY
PNG
CITATIONS
DOCS
PK DOC_ID
JOURNAL
YEAR
VOLUME
ISSUE
FIRST_PAGE
LAST_PAGE
PUBMED_ID
EXPERIMENTAL DATA
ACTIVITIES
PK ACTVITY_ID
ASSAY_ID
DOC_ID
MOLREGNO
RECORD_ID
ACTIVITY_TYPE
RELATION
PUBLISHED_VALUE
PUBLISHED_UNITS
STANDARD_VALUE
STANDARD_UNITS
ACTIVITY_COMMENT
ASSAYS
PK ASSAY_ID
ASSAY_TYPE
DOC_ID
DESCRIPTION
ASSAY2TARGETS
PK ASSAY_ID
TID
ASSAY_TAX_ID
ASSAY_STARIN
RELATIONSHIP_TYPE
CONFIDENCE
MULTI
COMPLEX
ASSAY_ORGANISM
TARGETS
TARGET_DICTIONARY
PK TID
TARGET_TYPE
DB_SOURCE
DB_VERSION
PROTEIN_ACCESSION
DESCRIPTION
GENE_NAMES
PREF_NAME
SYNONYMS
KEYWORDS
PROTEIN_SEQUENCE
PROTEIN_MD5SUM
TAX_ID
ORGANISM
TISSUE
STRAIN
CELL_LINE
IN_DRUGSTORE
IN_STARLITE
TABLE
PK TC_ID
FK TID
L1
L2
L3
L4
L5
L6
L7
L8
TARGET_CLASSIFICATION
ASSAY_TYPE
PK ASSAY_TYPE
ASSAY_DESC
RELATIONSHIP_TYPE
PK RELATIONSHIP_TYPE
RELATIONSHIP_DESC
TARGET_TYPE
PK TARGET_TYPE
TARGET_DESC
CONFIDENCE_LOOKUP
PK CONFIDENCE
CONFIDENCE_SUMMARY
DESCRIPTION
VERSION
PK NAME
CREATION_DATE
COMMENTS
LOOKUP TABLES
