# Supplementary figures and images for: Fifteen years of ChEMBL and its role in cheminformatics and drug discovery
Source: J Cheminform. 2025 Mar 10;17:32. doi: 10.1186/s13321-025-00963-z (PMC11895189; doi:10.1186/s13321-025-00963-z)

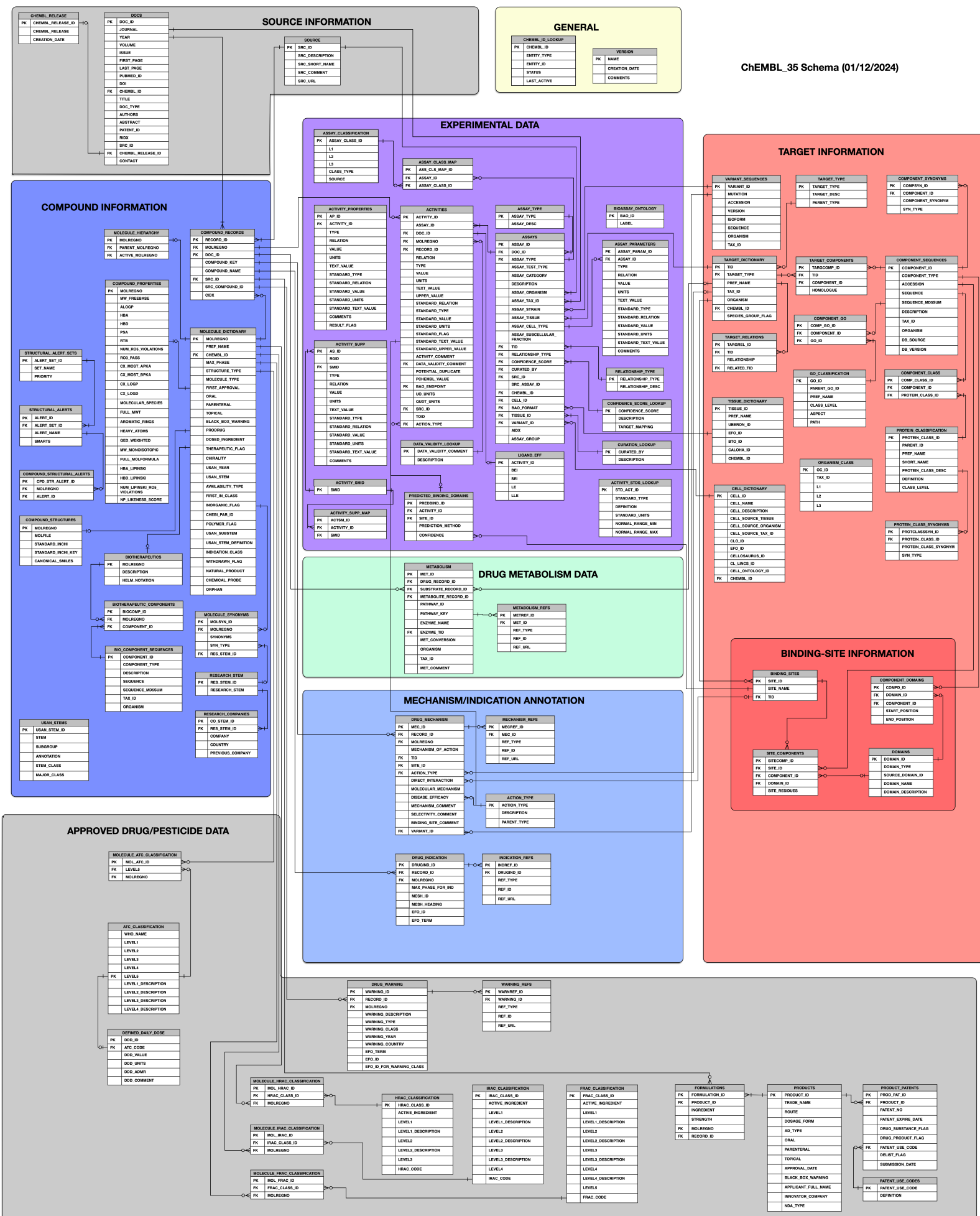

Supplement: Supplementary file 2 — Supplementary Material 2: Fig. 2: Entity–relationship diagram for ChEMBL 35. [file 13321_2025_963_MOESM2_ESM.pdf]
